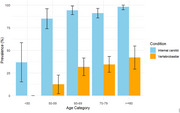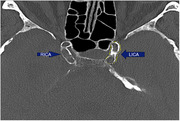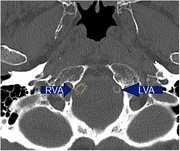# Prevalence, volume, and clinical correlates of intracranial arteriosclerosis among patients with cognitive complaints: a clinic‐based study

**DOI:** 10.1002/alz70860_106333

**Published:** 2025-12-23

**Authors:** OLIYAD M. MERDASSA, Francesco U S Mattace Raso, Meike W. Vernooij, Tavia Evans, Esther Van den Berg, Daniel Bos, Frank J. Wolters

**Affiliations:** ^1^ Erasmus MC ‐ University Medical Center, Rotterdam, South Holland, Netherlands; ^2^ Erasmus MC University Medical Center, Rotterdam, Netherlands; ^3^ Department of Epidemiology, Erasmus University Medical Center, Rotterdam, Netherlands; ^4^ Erasmus University Medical Center, Rotterdam, Netherlands; ^5^ Erasmus MC, Rotterdam, Netherlands; ^6^ Department of Radiology and Nuclear Medicine, Erasmus University Medical Center, Rotterdam, Netherlands; ^7^ European Society of Neuroradiology, Regensdorf, Switzerland; ^8^ Erasmus University Medical Center, Rotterdam, South Holland, Netherlands; ^9^ Department of Cardiovascular Sciences, Leuven, Belgium; ^10^ Department of Epidemiology, Havard TH Chan School of Public Health, Boston, MA, USA; ^11^ Harvard T.H. Chan School of Public Health, Boston, MA, USA; ^12^ Erasmus MC ‐ University Medical Center Rotterdam, Rotterdam, Zuid‐Holland, Netherlands; ^13^ Department of Radiology & Nuclear Medicine and Alzheimer Center, Erasmus MC, Rotterdam, Netherlands; ^14^ Department of Epidemiology, Erasmus MC, Rotterdam, Netherlands

## Abstract

**Background:**

Intracranial arteriosclerosis increases the risk of dementia in the general population, but no published studies have determined its clinical value for the diagnosis of vascular cognitive impairment in a memory clinic setting.

**Method:**

We included 317 patients (mean age: 69.9 years, 44% female) who attended the Erasmus MC Alzheimer Center between 2016 and 2021, and underwent non‐contrast multi‐detector computed tomography (MDCT) scans as part of their diagnostic workup. We determined the prevalence and volume of intracranial artery calcification in the internal carotid artery and vertebrobasilar arteries, as a marker of intracranial arteriosclerosis, and compared these to prevalence and volume in an age‐ and sex‐matched sample of the population‐based Rotterdam Study. Finally, we assessed cognitive function using comprehensive neuropsychological tests and determined the explained variance in cognitive performance when adding intracranial calcification to a model including age, sex and cardiovascular risk factors.

**Result:**

Overall prevalence of intracranial artery calcification was 89.91% (95%CI: 85.92‐92.89%), higher in the internal carotid artery (89.3% [85.9%–92.7%] than in the vertebrobasilar arteries (29.3% [24.3%–34.3%]). Prevalence of calcification was higher in women than in men in the internal carotid artery, while men ‐if affected‐ had larger volumes of calcification. Prevalence of both anterior and posterior circulation calcification was higher among memory clinic patients than in the age‐ and sex‐matched reference population (internal carotid artery: OR [95%CI] 2.22 [1.24–3.97]; and vertebrobasilar arteries: OR 1.40 [1.04–1.89]). Analysis of cognitive function will be presented at the conference.

**Conclusion:**

The vast majority of patients attending a memory clinic have evidence of intracranial arteriosclerosis, with higher prevalence and volume of calcification than age and sex‐matched reference participants in the general population. In ongoing analyses we explore the utility of intracranial arteriosclerosis imaging for the diagnosis of vascular cognitive impairment.